# The Anti-Biofilm Efficacy of Caffeic Acid Phenethyl Ester (CAPE) *In Vitro* and a Murine Model of Oral Candidiasis

**DOI:** 10.3389/fcimb.2021.700305

**Published:** 2021-08-02

**Authors:** Patrícia Pimentel de Barros, Rodnei Dennis Rossoni, Maíra Terra Garcia, Valéria de Lima Kaminski, Flávio Vieira Loures, Beth Burgwyn Fuchs, Eleftherios Mylonakis, Juliana Campos Junqueira

**Affiliations:** ^1^Department of Biosciences and Oral Diagnosis, Institute of Science and Technology, São Paulo State University (UNESP), São José dos Campos, Brazil; ^2^Multicampi School of Medical Sciences, Federal University of Rio Grande do Norte (UFRN), Caico, Brazil; ^3^Applied Immunology Laboratory, Institute of Science and Technology, Federal University of São Paulo (UNIFESP), São José dos Campos, Brazil; ^4^Division of Infectious Diseases, Rhode Island Hospital, Warren Alpert Medical School at Brown University, Providence, RI, United States

**Keywords:** CAPE (caffeic acid phenethyl ester), *Candida albicans*, Biofilms, *Galleria mellonella*, gene expression, oral candidiasis, β-defensin 3

## Abstract

*Candida albicans* is the main fungal species associated with the development of oral candidiasis. Currently, therapeutic options for these infections are limited by the adverse effects of antifungal drugs and by the emergence of drug resistant strains. Thus, the development of new antifungal agents is needed for the prevention and treatment of oral *Candida* infections. Caffeic acid phenethyl ester (CAPE) is a natural compound from propolis polyphenolic groups that exhibits many pharmacological properties. In this study, we investigated whether CAPE can have antifungal and immunomodulatory effects on oral candidiasis. Preliminary tests to assess the antifungal activity of CAPE were performed using the Minimum Inhibitory Concentration (MIC) assay that demonstrated inhibition in a range from 16 to 32 μg/mL, confirming its antifungal activity on several *C. albicans* strains isolated from the oral cavity. Subsequently, we analyzed *Candida* spp biofilms formed *in vitro*, in which CAPE treatment at 5 x MIC caused a reduction of 68.5% in the total biomass and ~2.60 Log in the viable cell count (CFU/mL) in relation to the untreated biofilm (*p*<0.0001). Next, RNA was extracted from untreated and CAPE-treated biofilms and analyzed by real-time qPCR. A series of genes analyzed (*ALS1, ECE1, EPA1, HWP1*, *YWP1, BCR1, BGR1, CPH1, EFG1*, *NDT80, ROB1, TEC1*, *UME6*, *SAP2*, *SAP5*, *PBL2*, and *LIP9*) were downregulated by CAPE compared to the untreated control group (*p*<0.0001). In *in vivo* studies using *Galleria mellonella*, the treatment with CAPE prolonged survival of larvae infected by *C. albicans* by 44.5% (*p* < 0.05) and accompanied by a 2.07-fold increase in the number of hemocytes. Flow cytometry revealed the most prominent increases were in types P2 and P3 hemocytes, granular cells, which phagocytize pathogens. In addition, CAPE treatment decreased the fungal load in the hemolymph and stimulated the expression of antifungal peptide genes such as *galiomicin* and *gallerimycin*. The antifungal and immunomodulatory activities observed in *G. mellonella* were extended to a murine model of oral candidiasis, in which CAPE decreased the levels of *C. albicans* colonization (~2 log CFU/mL) in relation to the untreated control group. In addition, CAPE treatment significantly reduced pseudomembranous lesions, invasion of hyphae on epithelium surfaces, tissue damage and inflammatory infiltrate (*p* < 0.05). CAPE was also able to increase the expression of *β-defensin 3* compared to the infected and untreated group by 3.91-fold (*p* < 0.0001). Taken together, these results show that CAPE has both antifungal and immunomodulatory effects, making it a promising natural antifungal agent for the treatment and prevention of candidiasis and shows impact to oral candidiasis.

## Introduction

*Candida albicans* is the most prevalent fungus in the human oral mycobiome (Bandara et al., 2019) and can be a harmless commensal organism or, upon appropriate conditions, transition to a pathogen. It possess several virulence factors that can lead to overgrowth and invasion of host tissues, causing a range of intraoral, pharyngeal, and perioral manifestations that determine different forms of oral candidiasis (Lewis and Williams, 2017; Hellstein and Marek, 2019; Ponde et al., 2021). Some of the most important virulence factors of *C*. *albicans* are the expressions of adhesins, the secretion of hydrolytic enzymes, its ability to transition from yeasts to hyphal and the high metabolic adaptability (Ciurea et al., 2020). Perhaps one of the most significant transition events is the formation of biofilms, cellular communities that can adhere and proliferate in abiotic and biotic surfaces. Oral *Candida* biofilms are more resistant to standard antifungal treatments and present a higher relapse rates, which turn these infections into a considerable clinical challenge in dental practice that are difficult to eradicate (Ponde et al., 2021).

In addition to therapeutic challenges posed by biofilm drug recalcitrance, the current therapeutic options for oral candidiasis are limited due to few availability antifungal classes, undesirable side effects, and antifungal resistance (Campoy and Adrio, 2017; Scorzoni et al., 2021). Thus, the search for new compounds that can control *Candida* spp. infections and avoid the aforementioned issues had pointed to natural compounds that display antifungal efficacy comparable to or stronger than drugs currently available for clinical use (Ferreira et al., 2021; Scorzoni et al., 2021). In this context, caffeic acid phenethyl ester (CAPE) stands out as a potent and promising agent that appears to modulate host responses (Breger et al., 2007) and may have a better impact on treating oral candidiasis.

CAPE is one of the bioactive compounds of propolis that has several biological and pharmacological properties, including antioxidant, anti-inflammatory, anticarcinogenic, anti-viral, antifungal and immunomodulatory activities (Breger et al., 2007; Chan et al., 2013; Armutcu et al., 2015; Coleman et al., 2016). CAPE modulates the transcription factors NF-kβ showing an anti-inflammatory activities (Armutcu et al., 2015; Olgierd et al., 2021). The NF-κβ pathway is involved in the transcription of many cytokines, chemokines, enzymes, antiapoptotic and cell growth factors (Armutcu et al., 2015; Olgierd et al., 2021), signaling involved in a wide range of physiological processes throughout the body, including immune responses, cell proliferation and inflammation (Natarajan et al., 1996).

Thus, the aim of this study was to evaluate the antifungal and immunomodulatory effects of CAPE on *C. albicans* using *in vitro* and *in vivo* models. *In vitro* assessment focused on the fungal biofilm morphology that contributes to *C. albicans* virulence and is challenging to treat. CAPE efficacy was further assessed *in vivo* using *G. mellonella* and a murine oral candidiasis model.

## Materials and Methods

### Microorganisms and Growth Conditions

Forty oral clinical strains of *Candida albicans* and a reference strain (ATCC18804) from the fungal collection of the Oral Microbiology and Immunology Laboratory at the Institute of Science and Technology of São José dos Campos/UNESP were used in this study (Junqueira et al., 2012). The strains were cultured in yeast extract, peptone, dextrose (YPD broth, Difco, Detroit, USA) for 24 h at 37°C.

### Antifungal Activity and Determination of Minimum Inhibitory Concentration (MIC)

All clinical *C. albicans* strains were subjected to broth microdilution assays to determine the MIC values for CAPE, amphotericin B and fluconazole. Quality control was performed using the strain *C. parapsilosis* ATCC 22019.

Investigational compounds CAPE, amphotericin B and fluconazole were purchased from Sigma/Aldrich and diluted in DMSO at a concentration of 10 mg/mL. MICs were determined according to Clinical and Laboratory Standards Institute, document M60 (Clinical Laboratory Standard Institute, 2017) using round-bottomed 96-well microtiter plates, containing *Candida* suspensions prepared in physiological solution and then diluted into RPMI 1640 at final concentrations of 0.5-2.5 x10^3^ CFU/mL. Inoculated plates were incubated for 24-48 h at 37°C. Fluconazole and amphotericin B were used as positive controls with final concentrations ranging from 64 to 0.125 μg/mL. The concentration tested for CAPE ranged from 128 to 2 µg/mL. The breakpoints used followed the CLSI guidelines (Fluconazole: ≤ 2.0 μg/mL for susceptible, ≥ 8 μg/mL for resistant; Amphotericin B: >1 μg/mL for resistant) (CLSI, 2017). MIC values for fluconazole and amphotericin B were defined as the lowest concentrations that inhibited 50% and 90% of fungal growth, respectively.

### *In Vitro* Study in *C. albicans* Biofilms

The anti-biofilm activity of CAPE was tested against two *C. albicans* strains resistant to fluconazole (CA14 and CA70)*. C. albicans* pre-biofilms were formed in 96-well flat bottom microtiter plates (TPP^®^, Trasadingen, Switzerland), following the methodology described by de Barros et al. (2018). Briefly, standardized suspensions of *C. albicans* (10^7^ cells/mL) were added in microtiter plates containing yeast nitrogen base broth (YNB) (Difco, Detroit, USA) with 100 mM glucose and incubated for 48 h at 37°C with shaking at 75 rpm. After this period, each well was washed twice with PBS, and 200 µL of YNB containing different concentrations of CAPE (2 x MIC and 5 x MIC) were added to the plates and incubated for another 24 h in the same experimental conditions. After the treatment, each well was washed twice with PBS for subsequent analysis of viability cell and total biomass. Fluconazole served as a positive control, in concentrations corresponding to 2 x MIC and 5 x MIC.

### Biofilm Analysis by Viability Assay

For the biofilm viability assay, 200 µL of PBS was added to each well and the biofilms were disrupted using a ultrasonic homogenizer (Sonopuls HD 2200, Bandelin Electronic, Berlin, Germany) at 50 W for 30 s (de Barros et al., 2018). Serial dilutions were prepared and 10 µL aliquots of the dilutions were plated on Sabouraud Dextrose agar supplemented with chloramphenicol. The plates were incubated at 37°C for 48 h and the number of colony-forming units (CFU/mL) was counted. Three biological replicates were performed, using five biofilms per group.

### Biofilm Analysis by Biomass Quantification

Biofilm biomass was quantified by crystal violet (CV) staining as described by (Rossoni et al., 2018). After washing the wells, they were incubated for 15 min with 100 μL of 99% methanol (Sigma-Aldrich, St. Louis, MO, USA) followed by 20 min of 1% CV solution and washed with 0.85% NaCl. Bound CV was released by adding 150 μL of 99% methanol and the absorbance was measured at 570 nm (AJX-1900 spectrophotometer, Micronal, São Paulo, Brazil) to determine the total amount of biofilm dyed with CV. The experiments were performed three times, using five biofilms per group.

### Biofilm Analysis by Scanning Electron Microscopy (SEM)

Biofilms formed on resin discs were fixed in 1 mL of 2.5% glutaraldehyde for 1 h (de Barros et al., 2018). Specimens were dehydrated in 100% ethanol (Sigma-Aldrich, St. Louis, MO, USA) for 20 min. The plates were kept in an incubator at 37°C for 24 h to permit total drying of the specimens. After drying, the specimens were transferred to aluminum stubs and sputter coated with gold for 160 s at 40 mA (Denton Vacuum Desk II, Denton Vacuum LLC, Moorestown, NJ, USA). Images were acquired using a JEOL JSM-5600 scanning electron microscope (JEOL USA, Inc., Peabody, MA, USA).

### Expression Analysis of *C. albicans* Biofilm Genes

Biofilms formed in 24-well microtiter plates, as described in section 2.3, were used to assess transcription of *C. albicans* pathogenicity-associated genes (de Barros et al., 2017). After incubation, 1 mL TRIzol^®^ (Ambion, Carlsbad, CA, USA) was added to each well to extract RNA according to the manufacturer’s protocol. RNA concentration was measured using a NanoDrop 2000 Spectrophotometer (Thermo Fisher Scientific, Wilmington, DE, USA). Total extracted RNA (700 ng) was treated with RQ1 RNase-Free DNase (Promega Corporation, Madison, WI, USA) and transcribed to cDNA using the GoScript™ Reverse Transcription Mix, Random Primers (Promega Corporation, Madison, WI, USA) according to the manufacturer’s recommendations. Primers for the genes analyzed are listed in **Table 1**. cDNA was amplified to determine the relative quantification of *ALS1, ECE1, EPA1, HWP1*, *YWP1*, *BCR1, BGR1, CPH1, EFG1*, *NDT80, ROB1, TEC1*, *UME6*, *SAP2*, *SAP5*, *PBL2* and *LIP9* expression. Three reference genes, *ACT1*, *PMA1* and *RIP1*, were tested in all experimental groups. The results were analyzed at http://www.leonxie.com/referencegene.phpe, and the selected reference gene was *RIP1* (data not shown).

**Table 1:** Quantitative real-time PCR primers.

Candida albicans				
Target gene	Oligonucleotide sequence 5’–3’*	Description	Amplicon size (bp)**	Reference
*ACT1*	**F**- GAAGCCCAATCCAAAAGA **R**- CTTCTGGAGCAACTCTCAATTC	Structural constituent of cytoskeleton (Normalizing internal standard)	130 bp	Nailis et al., 2010
*RIP1*	**F**- TGTCACGGTTCCCATTATGATATTT **R**- TGGAATTTCCAAGTTCAATGGA	Ubiquinol cytochrome-c reductase complex component (Normalizing internal standard)	83 bp	Nailis et al., 2010
*PMA1*	**F**- TTGCTTATGATAATGCTCCATACGA **R**- TACCCCACAATCTTGGCAAGT	Plasma Membrane H (+) ATPase (Normalizing internal standard)	66 bp	Nailis et al., 2010
*ALS1*	**F**- CAACAGGCACCTCAGCATCTAC **R**- CTCCACCAGTAACAGATCCACTAGTAA	Agglutinin-Like Sequence (cell adhesion molecule binding)	88 bp	Nailis et al., 2010
*BCR1*	**F**- AATGCCTGCAGGTTATTTGG **R**- TTTTAGGTGGTGGTGGCAAT	Biofilm and Cell wall Regulator (transcription factor activity)	112 bp	Hnisz et al., 2012
*BGR1*	**F**- ACGATCAACCATTAGTGGAGG **R**- GAAGAAGTAGGTGTAGATGATCCAC	Biofilm ReGulator (transcription factor activity)	141 bp	Hnisz et al., 2012
*CPH1*	**F**- ACGCAGCCACAAGCTCTACT **R**- GTTGTGTGTGGAGGTTGCAC	*Candida* pseudohyphal regulator (transcription factor activity)	119 bp	Sherry et al., 2014
*EFG1*	**F**- CAGTATGGTCAGTATAATGCT **R**- TGTTGTTGCTGTTGGTATGGATATGATGATG	Enhanced filamentous growth (transcription factor activity)	222 bp	Hnisz et al., 2012
*ECE1*	**F-** GTCGTCAGATTGCCAGAAATTG **R-** CTTGGCATTTTCGATGGATTGT	Extent of Cell Elongation (Candidalysin)	69 bp	Bassilana et al., 2005
*EPA1*	**F-** ACCACCACCGGGTATACAAA **R-** GCCATCACATTTGGTGACAG	Epithelial adhesion protein (adhesion)	99 bp	Sherry et al., 2014
*HWP1*	**F**- GAAACCTCACCAATTGCTCCAG **R**- GTAGAGACGACAGCACTAGATTCC	Hyphal wall protein (cell adhesion molecule binding)	92 bp	Hnisz et al., 2012
*NDT80*	**F-**CTCAACAAGGCCCAACACCTC **R-** TTGACGTGGTTGTCTTGCTGG	Meiosis-specific transcription factor	121 bp	Hnisz et al., 2012
*ROB1*	**F-** GAGGAATTTTACGCCCAACA **R-** TCTTGTGGTTGTGGTTCGTC	Regulator of Biofilm	130 bp	Hnisz et al., 2012
*TEC1*	**F-** TGAGCAACAACAACAACAACCAC **R-** CTGGGTTGTTGTCATAGTGGCC	TEA/ATTS transcription factor	140 bp	Hnisz et al., 2012
*UME6*	**F**- TCATTCAATCCTACTCGTCCACC **R**- CCAGATCCAGTAGCAGTGCTG	Zn(II)2Cys6 transcription factor	133 bp	Hnisz et al., 2012
*SAP2*	**F-** GAATTAAGAATTAGTTTGGGTTCAGTTGA **R-**CCACAAGAACATCGACATTATCAGT	Major secreted aspartyl proteinase	122 bp	Nailis et al., 2010
*SAP5*	**F-** CCAGCATCTTCCCGCACTT **R-** GCGTAAGAACCGTCACCATATTTAA	Secreted Aspartyl Proteinase	96 bp.	Nailis et al., 2010
*PLB2*	**F-** TGAACCTTTGGGCGACAACT **R-** GCCGCGCTCGTTGTTAA	PhosphoLipase B	80 bp	Nailis et al., 2010
*LIP9*	**F-** CGCAAGTTTGAAGTCAGGAAAA **R-** CCCACATTACAACTTTGGCATCT	LIPase	119 bp	Nailis et al., 2010
*YWP1*	**F**- ACACCGGAAAATACCGTTGC **R**- ATGGCAGCTTTACCAGAACC	Yeast-form wall protein (adhesion of symbiont to host)	116 bp	Granger, 2012
*Galleria mellonella*				
***β*** *-actin*	**F**- ACAGAGCGTGGCTACTCGTT **R**-GCCATCTCCTGCTCAAAGTC	Structural constituent of cytoskeleton (Normalizing internal standard)	104 bp	Rossoni et al., 2017
***Galiomicin***	**F**- TCCAGTCCGTTTTGTTGTTG **R**- CAGAGGTGTAATTCGTCGCA	Antifungal peptide	123 bp	Rossoni et al., 2017
***Gallerymicin***	**F**- GAAGATCGCTTTCATAGTCGC **R**- TACTCCTGCAGTTAGCAATGC	Antifungal peptide	175 bp	Bergin et al., 2006
*Mus Musculus*				
***GAPDH***	**F**- CGTCCCGTAGACAAAATGGT **R**- GAATTTGCCGTGAGTGGAGT	Glyceraldehyde-3-phosphate dehydrogenase (Normalizing internal standard)	177 bp	Tomalka et al., 2015
***β*** *defesin3*	**F**- GCATTGGCAACACTCGTCAGA **R**- CGGGATCTTGGTCTTCTCTATT	Beta-defensin 3	85 bp	Tomalka et al., 2015

*F- Forward and **R**- Reverse.

**bp- base pairs.

For the qPCR reactions, a GoTaq^®^ qPCR Master Mix kit (Promega Corporation, Madison, WI, USA) was used according to the manufacturer’s recommendations. The reactions were performed in triplicate in the StepOnePlus™ Real-Time PCR System (Applied Biosystems, Foster, CA, USA), consisting of 12.5 μL GoTaq^®^ qPCR Master, 0.5 μL CXR, 0.4 μM primers (final concentration), and target cDNA, supplemented with RNAse-free ddH_2_O to a final volume of 25 μL. The cycling parameters for the amplification reactions were 95°C for 2 min; followed by 40 cycles of 95°C for 15 s and 60°C for 30 s. The level of gene expression was calculated by applying the 2^-ΔΔCT^ method (Livak and Schmittgen, 2001).

### *In Vivo S*tudy in *G. mellonella* Model

The antifungal and immunomodulatory effects of CAPE on *G. mellonella* model were assessed in larvae infected with the *C. albicans* 70 strain (resistant to fluconazole). For this study, the methodology described by Rossoni et al. (2019) was used with some modifications. *G*. *mellonella* in their final larval stage (250-350 mg) were stored in the dark and used within seven days from shipment. Groups inoculated with PBS and without any intervention (naïve) were included in the assays as controls.

*G. mellonella* larvae were infected with *C. albicans* and treated with CAPE 30 min after inoculation. At 24 h post-treatment, the experimental candidiasis was monitored by survival rate, hemocytes count, characterization of the hemocytes population by flow cytometry, fungal load in the hemolymph, and gene expression of antifungal peptides.

### *G. mellonella* Survival

Initially, CAPE toxicity was evaluated. The compound was injected at varying concentrations (5-20 mg/kg) directly into *G. mellonella* hemolymph and monitored for 7 days to generate survival curves. To study the CAPE effects on candidiasis, larvae were infected with 5 x 10^7^ cells/mL of *C. albicans*, and injected with CAPE (5 mg/kg) or fluconazole (5 mg/kg) after 30 min inoculation. Larvae were considered dead when they did not respond to touch. Sixteen larvae were used for each group.

### *G. mellonella* Hemocyte Density

After 24 h of the treatments as described above, the hemolymph was collected into ice-cold sterile insect physiologic solution (IPS; 150 mM sodium chloride, 5 mM potassium chloride, 100 mM Tris hydrochloride, pH 6.9, with 10 mM EDTA, and 30 mM sodium citrate). After centrifugation, the supernatant was discarded, and the hemocytes were diluted in IPS medium and quantified using a hemocytometer. The results were averaged from four replicates.

### Characterization of Hemocytes Sub-Populations in *G. mellonella*

The hemocytes sub-populations were analyzed by Fluorescence-Activated Cell Sorting (FACS). For this, hemolymph (150 mL) was extracted from larvae and diluted in ice cold PBS (800 μL) (Browne et al., 2015). Hemocytes were enumerated and the density was adjusted to 1 x 10^6^ cells/mL. Cells were fixed in 4% formaldehyde (Sigma-Aldrich) in PBS for 10 min at 4°C. Hemocytes were washed in 1% BSA/PBS, collected through centrifugation at 1500 x g for 5 min at 4°C and resuspended in BSA/PBS at a density of 1 x 10^6^ cells/mL. Cell populations were characterized using a FACSLyric™ flow cytometer (BD Biosciences) and differentiated based on side and forward scatter with a total of 10,000 events measured per sample using the BD FACSuit™ software. Hemocytes sub-populations were gated and analyzed using FlowJo™ Software.

### Analysis of the Gene Expression of Antifungal Peptides in *G. mellonella*

The gene expression assay was performed as described by de Barros et al. (2019). Larvae were pulverized using a liquid nitrogen cooled mortar and pestle. RNA was extracted according to the manufacturer’s instruction using TRIzol (Ambion, Inc., Carlsbad, CA, USA). For cDNA preparation, the extracted total RNA (1000 ng) was transcribed applying reverse transcriptase and random primers according to the manufacturer’s instructions (GoScript Reverse Transcription Mix, Random Primers, Promega Corporation, Madison, WI, USA). Primers for the genes examined in this study were described in **Table 1**. The transcribed cDNAs were amplified for relative quantification of the expression of the genes encoding *gallerimycin* and *galiomicin* in relation to the concentration of the reference gene *β-actin*. Quantitative real-time PCR was conducted using the GoTaq qPCR Master Mix Kit (Promega Corporation, Madison, WI, USA) in the StepOnePlus™ apparatus (Applied Biosystems, Framingham, MA, USA). The level of gene expression was calculated by applying the 2^-ΔΔCT^ method (Livak and Schmittgen, 2001).

### Quantification of *Candida* in *G. mellonella* Hemolymph

For this study, the methodology described by de Barros et al. (2019) was used with some modifications. Fungal cells were extracted from *G. mellonella* hemolymph immediately after treatment (0 h) and 24 h after the larvae were treated with of CAPE or fluconazole. Five larvae were used in order to collect a pool of hemolymph. The experiment was performed in triplicate and a total of 15 larvae were used for each group and period. The extracted hemolymph was homogenized and aliquots of 0.1 mL of each dilution were transferred to Petri plates containing Sabouraud Dextrose agar supplemented with chloramphenicol. The plates were incubated for 48 h at 37°C and colonies were subsequently counted to calculate the CFU/mL numbers.

### *In Vivo S*tudy in Murine OralCandidiasis Model

Adult 90-days-old male *Swiss* mice, weighing approximately 30g, from the Central Animal Care Facility of UNESP (Botucatu, SP, Brazil) were used in the study with approval from the Ethics Committee on the Use of Animals of the ICT/UNESP under protocol 019/2019-CEUA-ICT/UNESP. A total of 35 mice were divided into the following groups: mice not infected by *C. albicans* and untreated (n = 3) (control group), mice not infected by *C. albicans* and treated with CAPE (n = 8), mice infected by *C. albicans* and untreated (n = 8), mice infected by *C. albicans* and treated with nystatin (n = 8), and mice infected by *C. albicans* and treated with CAPE (n = 8). The control group was used as parameter to evaluate histological alterations and baseline expression of the genes investigated.

Oral candidiasis was induced as previously described by de Camargo Ribeiro et al. (2020). Animals were immunosuppressed using prednisolone (Depo-Medrol, Laboratórios Pfizer Ltda., Guarulhos, Brazil) on alternating days with *Candida* inoculations. To decrease normal oral bacteria, tetracycline hydrochloride (0.83mg/mL, Terramycin, Pfizer) was administered in the drinking water during the experiment period. For *C. albicans* infection, animals were anesthetized with intraperitoneal injections of ketamine (100 mg/kg) and xylazine (10 mg/kg) and inoculated with a *C. albicans* suspension (10^9^ cells/mL) using a sterile swab. After 24 h of the second infection, 200 μL of the CAPE at a dose of 15 mg/kg or nystatin (40,000 IU/kg in 0.2 ml PBS) (Matsubara et al., 2012) were delivered into the oral cavity using a pipette.

### Recovery of *C. albicans* From the Oral Cavity of Mice

After 48 h of treatment, saliva samples were recovered by mouthwash using 100 µL of sterile PBS. The samples were diluted and plated on Sabouraud Dextrose (SD) agar with chloramphenicol to determine the number of CFU/mL.

### Macroscopic Analysis of Oral Candidiasis

After 48 h of treatment, euthanasia was performed and the dorsum tongue of the mice were analyzed on a stereomicroscopy (Zeiss, Göttingen, Germany) and scored between 0-4 for the presence of lesions. The scoring system was as follows: 0) absence of lesions; 1) white plaques on less than 20% of the tongue surface; 2) white plates covering 21-90% of the surface; 3) white plaques by more than 91%; 4) thick lesions with pseudomembrane in more than 91% (Takakura et al., 2003; de Camargo Ribeiro et al., 2020).

### Microscopic Analysis of Oral Candidiasis

For the microscopic analysis, the tongues were removed, sagittally sectioned and fixed in 10% formalin for 24 h. After paraffin embedding, 5 μm serial sections were obtained, and stained with Hematoxylin-Eosin (HE) and Schiff’s Periodic Acid (PAS) according to laboratory standard protocols. On the histologic sections it was evaluated the presence of yeasts, hyphae and candidiasis lesions. The intensity of candidiasis lesions was evaluated following the study of Junqueira et al. (2005), considering epithelial alterations and inflammatory response of the conjunctive tissue.

### Analysis of *β-defensin 3* Gene Expression in the Tongue of Mice

The analysis of *β-defensin 3* expression assay was performed as described by Tomalka et al. (2015). Frozen tongue was homogenized in 1 mL of TRIzol (Ambion, Inc., Carlsbad, CA, USA) in a porcelain mortar using a pestle and RNA extraction was made as recommended by the manufacturer. Total extracted RNA (1000 ng) was treated with RQ1 RNase-Free DNase (Promega Corporation, Madison, WI, USA) and transcribed to cDNA using the GoScript™ Reverse Transcription Mix, Random Primers (Promega Corporation, Madison, WI, USA). Relative quantification of gene expression (*β-defensin 3*) was determined by real-time PCR with SYBR green (GoTaq qPCR Master Mix Kit, Promega Corporation, Madison, WI, USA) normalized to *GAPDH.* These primers were described in **Table 1**. The level of gene expression was calculated by applying the 2^-ΔΔCT^ method (Livak and Schmittgen, 2001).

### Statistical Analysis

For statistical analysis level of significance of 5% was adopted. Analysis of variance (ANOVA), Tukey test, Student’s t-test, and Kruskal-Wallis were used depending on data distribution and groups, for each experimental assay. All analysis were performed using the GraphPad Prism 6 Program (GraphPad Software, Inc., La Jolla, CA, USA).

## Results

### Antifungal Activity of CAPE on *C. albicans* Clinical Strains

The antifungal effects of CAPE were evaluated on *C. albicans* growth by the minimum inhibitory concentration (MIC). Among the 40 clinical strains of *C. albicans* tested, 5 (12.5%) and 8 (20%) showed resistance to amphotericin B and fluconazole, respectively (**Table 2**). These strains showed susceptibility profile ranging from 0.0625 to 2 µg/mL for amphotericin B and 0.5 to ≥ 64 µg/mL for fluconazole. The overall susceptibility to CAPE ranged from 16 to 64 µg/mL (**Table 2**). *C. albicans* strains (CA70 and CA14) with resistance to fluconazole were selected for subsequent assays.

**Table 2:** Minimum Inhibitory Concentration (MIC) of CAPE against *Candida albicans* isolates.

	Amphotericin B (µg/mL)	Fluconazole (µg/mL)	CAPE (µg/mL)
*C. albicans* ATCC 18804	0.125	2	32
*C. albicans* SC5314	0.0625	1	32
*C. albicans* 3	0.5	1	64
*C. albicans* 5	0.5	4	64
*C. albicans* 14	1	64	32
*C. albicans* 17	0.25	32	32
*C. albicans* 21	1	0.5	64
*C. albicans* 23	0.25	1	32
*C. albicans* 26	0.0625	1	32
*C. albicans* 28	1	1	64
*C. albicans* 31	0.25	0.5	16
*C. albicans* 38	1	1	64
*C. albicans* 40	0.25	2	>64
*C. albicans* 52	1	1	64
*C. albicans* 53	0.25	4	64
*C. albicans* 60	1	1	16
*C. albicans* 61	0.25	16	32
*C. albicans* 70	0.625	>64	16
*C. albicans* 73	0.625	>64	64
*C. albicans* 4S	2	8	16
*C. albicans* 24S	1	2	64
*C. albicans* 31S	0.5	4	32
*C. albicans* 36S	1	2	32
*C. albicans* 39S	0.5	16	32
*C. albicans* 57S	2	2	16
*C. albicans* 65S	1	1	32
*C. albicans* 66S	2	2	16
*C. albicans* 69S	0.25	1	32
*C. albicans* 75S	2	4	32
*C. albicans* 97S	1	32	32
*C. albicans* 109S	0.25	1	64
*C. albicans* 110S	0.5	2	32
*C. albicans* 115S	0.25	4	32
*C. albicans* 137S	1	0.5	32
*C. albicans* 160S	0.625	2	16
*C. albicans* 165S	0.5	4	16
*C. albicans* 193S	1	1	64
*C. albicans* 194S	0.25	1	16
*C. albicans* 197S	1	1	64
*C. albicans* 205S	2	0.5	16
*C. albicans* 228S	1	0.5	32
*C. albicans* 230S	0.5	1	64
*C. parapsilosis* ATCC 22019	0.25	1	32

### Effects of CAPE on *C. albicans* Biofilms

**Figure 1:**
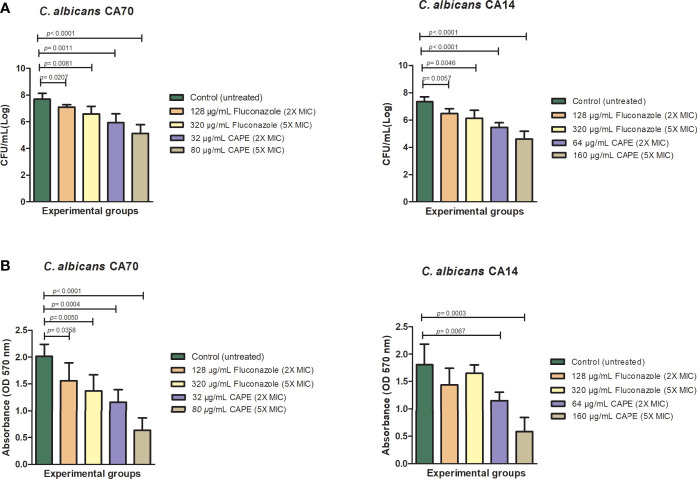
Analysis of in *vitro C. albicans* biofilms. Mature biofilms (48 h) formed in 96-wells plates for clinical strains of fluconazole-resistant *C. albicans* were treated with different concentrations of fluconazole and CAPE (2 x MIC and 5 x MIC). **(A)** The viable *C. albicans* cells number are shown in CFU per milliliter count. **(B)** Biofilm biomass analysis expressed in optical density (570nm). The treatments were compared to the control group using Student’s t test (*p* < 0.05).

Next, we investigated the activity of CAPE on *C. albicans* biofilms since this virulene factor has an important role in the recurrent and chronic infections. CAPE at 2 x MIC (32 µg/mL for CA70 and 64 µg/mL for CA14) and 5 x MIC (80 µg/mL for CA70 and 160 µg/mL for CA14) concentrations significantly reduced the viable cells number (*p*<0.0001) and the quantity of total biomass (*p*<0.0001) for both *C. albicans* strains. The higher fungal CFU/mL reductions observed corresponded to 2.57 and 2.74 log respectively for the strains CA70 and CA14, when the biofilms were treated with CAPE at 5 x MIC in relation to the untreated group. Significant reductions were also found in the treatment with fluconazole, however this antifungal was used in concentration of 320 µg/mL, which was 2 to 4 times higher than the CAPE concentrations (**Figure 1A**). In relation to the biofilm biomass, the treatment with CAPE and fluconazole at 5 x MIC caused a reduction of 68.5 and 32%, respectively, in relation to the untreated control group (**Figure 1B** **).**

**Figure 2:**
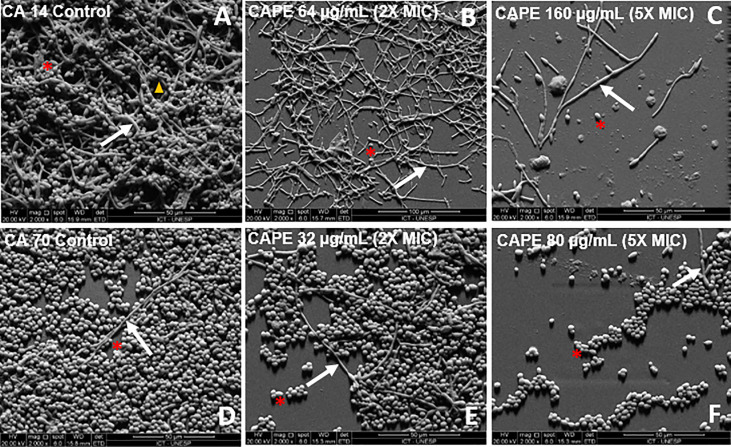
**(A–F)** Scanning electron microscopy images of biofilms formed in *vitro* on resin surfaces, as follows: biofilms formed by only *C. albicans* (CA14) **(A)** and CA70 **(D)**, cells treated with CAPE (2 x MIC) **(B, E)**, cells treated with CAPE (5 x MIC) **(C, F)**. It is possible to observe the presence of channel water (→), yeasts (*), and hyphae (▲) of *C. albicans*. Original magnification 2000x.

The effects of CAPE on *C. albicans* biofilms were visually confirmed by SEM. The SEM images revealed a dense biofilm, formed by several layers of hyphae associated with yeasts in the untreated control group (**Figures 2A, D**). However, the CAPE-treated groups exhibited biofilms formed by a single dispersed layer with fewer yeasts and hyphae (**Figures 2B, C, E, F**). These observations are consistent with the inhibition of biofilm observed in the viable cells count and total biomass assays.

**Figure 3:**
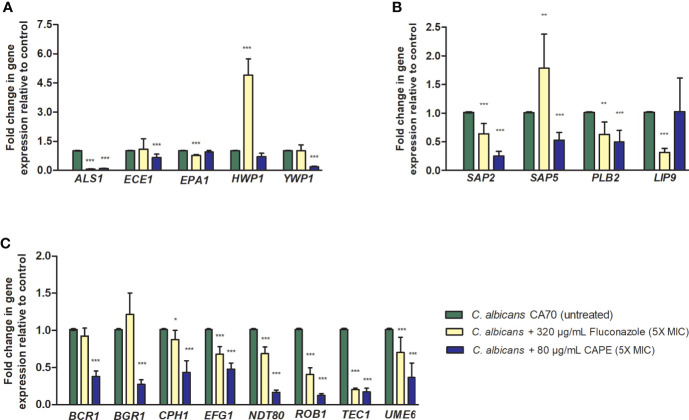
Relative expression of *C. albicans* genes. **(A)** Relative quantification of *ALS1, ECE1, EPA1, HWP1*, *YWP1* genes for the non-treated control group (PBS) and treated groups with fluconazole and CAPE (5 x MIC). **(B)** Relative quantification of *SAP2*, *SAP5*, *PBL2* and *LIP9* genes. **(C)** Relative quantification of *BCR1, BGR1, CPH1, EFG1*, *NDT80, ROB1, TEC1* and *UME6* genes. Normalization was done using *RIP1* gene and values expressed as the mean and SD. The treatments were compared to the control group using Student’s t-test (*p < 0.05, **p < 0.01 and ***p < 0.0001).

We also studied whether the inhibitory effect of CAPE on biofilm is related to changes in gene expression. To this end, the RNA was extracted from untreated and CAPE-treated biofilms and analyzed by real-time qPCR. Data obtained from real-time qPCR revealed a variety of *C. albicans* genes whose expression was affected differently upon CAPE treatment. CAPE at 5 x MIC downregulated the expression of genes involved in maintenance, development, and maturation of biofilms, such as transcriptional regulators: *BCR1, BGR1, CPH1*, *NDT80, ROB1 and TEC1* (2.6, 3.7, 2.32, 6.25, 8.33, 5.88-fold); adhesion: *ALS1, EPA1 and YWP1* (11.1, 1.06, 5.55-fold); and filamentation: *ECE1*, *HWP1*, *EFG1 and UME6* (1.53, 1.4, 2.08, 2.78-fold) when compared to control (**Figure 3**). Importantly, genes involved in *C. albicans* host invasion (*SAP2*, *SAP5*, *PBL2* and *LIP9*) were also downregulated 4.0, 1.92, 2.04, 0.98-fold, respectively, compared to the control group (*p* < 0.0001) (**Figure 3**). Conversely, *HWP1* and *SAP5* were upregulated with fluconazole treatment, increasing expression by 4.9 and 1.92-fold, respectively (*p* < 0.001) (**Figure 3**).

### Effects of CAPE on Experimental Candidiasis in *G. mellonella* Model

**Figure 4:**
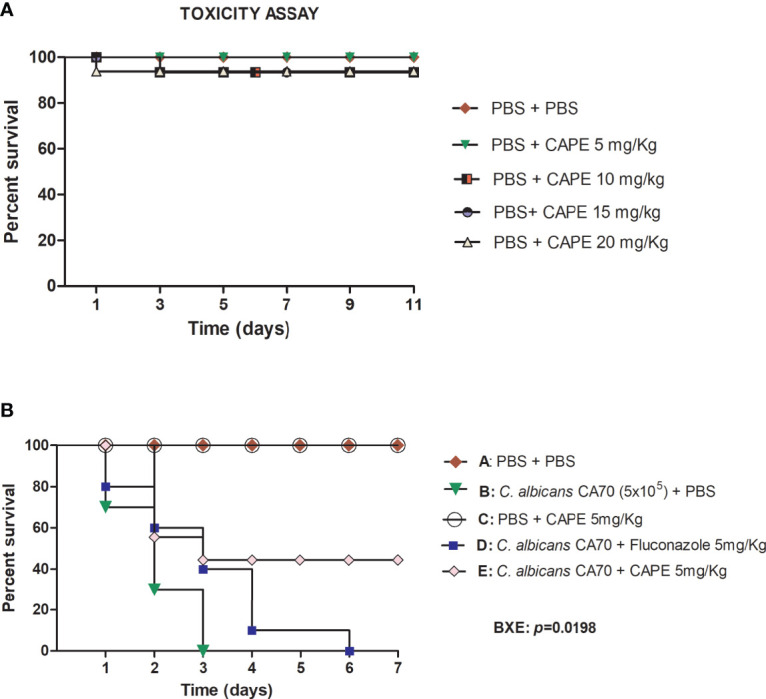
**(A)** Toxicity evaluation of CAPE in *G. mellonella* model. *G. mellonella* larvae were injected with serial concentrations of CAPE and no death was observed at the concentrations used. **(B)** CAPE prolonged the survival of *G. mellonella* larvae infected with *C. albicans* (CA70). Significant differences were observed in survival between the “CA70 + CAPE (5 mg/Kg) group” and “CA70 + PBS group”. Kaplan-Meier test, *p* ≤ 0.05.

Initially, the *G. mellonella* model was used to evaluate the toxicity of CAPE in single doses of 5, 10, 15 and 20 mg/kg. CAPE did not exert toxic effects on the larvae at the tested concentrations (**Figure 4A**). Next, *G. mellonella* model was used to study the efficacy of treatment with CAPE in larvae infected by *C. albicans*. In the untreated group, infection with *C. albicans* caused death in 100% of the larvae within 3 days. When the larvae were treated with CAPE (5 mg/kg) after *C. albicans* infection, the survival rate of *G. mellonella* larvae was significantly increased by 44.5% (*p* < 0.05). Conversely, treatment with fluconazole at a dose of 5 mg/kg killed 100% of the larvae within 6 days (**Figure 4B**). These results indicated that CAPE had *in vivo* efficacy in treating *C. albicans* infections without toxicity to the host at the delivered therapeutics concentration.

**Figure 5:**
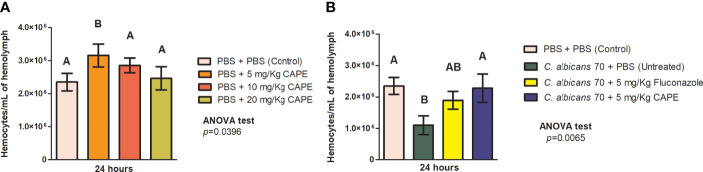
CAPE modulates the immune system of *G. mellonella*. **(A)** *G. mellonella* larvae were injected with serial concentrations of CAPE increased the hemocyte number compared to a PBS control (PBS + PBS). **(B)** The group of CA70 + CAPE (5 mg/Kg) increased significantly the hemocyte number compared to CA70 + PBS (untreated). CA70 + PBS group showed a reduction of hemocyte quantity in relation to the PBS control group, but when the larvae were treated with CAPE, the hemocyte quantity was very similar to the values found in the PBS control group. The number of hemocytes slightly increased in the group of CA70 + fluconazole (5 mg/Kg) but did not show differences in relation to the CA70 + PBS group. Different letters A and B represent statistically significant differences among the groups. ANOVA and Tukey Tests (*p* < 0.05 was considered significant).

To investigate the effects of CAPE on immune system of *G. mellonella* larvae, we evaluated the hemocytes quantity in the hemolymph of *G. mellonella* with or without *Candida* infection. Analyzing the larvae not infected by *C. albicans*, we observed a significant increase in the number of hemocytes in the group treated with CAPE at 5 mg/kg (1.3-fold increase) compared to the PBS control group (**Figure 5A**). When the larvae were infected by *C. albicans* and untreated, we found hemocytes reductions (2.2-fold decrease) compared to the control group. Larvae infected and treated with CAPE exhibited an increase in the number of hemocytes compared to the infected and untreated group. The treatment with fluconazole also led to an increase in the hemocytes quantity (1.71-fold increase) although it was less pronounced than the treatment with CAPE (2.07-fold increase) (**Figure 5B**). These data suggested that CAPE can influence the immune system, acting as an immunomodulatory natural compound.

**Figure 6:**
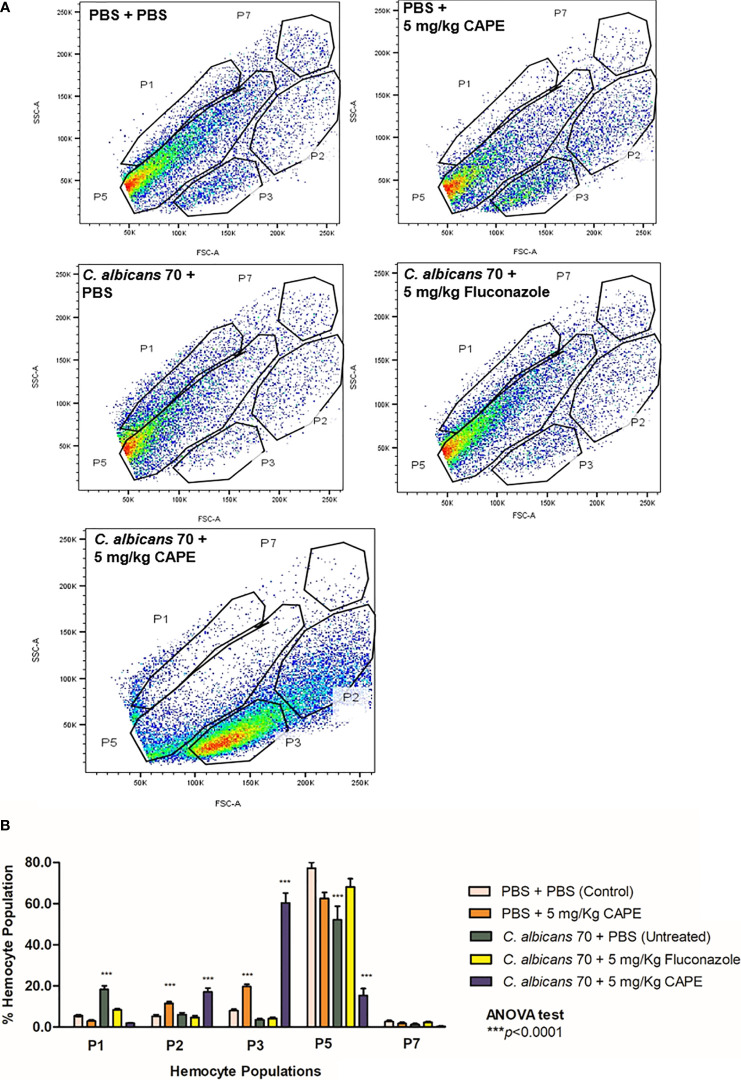
FACS analysis of larval hemocyte population. **(A)** Hemocytes were extracted from larvae and differentiated by FACS based on cell size (x-axis) and granularity (y-axis). Representative images of hemocytes in the P1, P2, P3, P5, and P7 sub-populations are presented in each group. **(B)** Fluctuations in hemocyte sub-populations in larvae for the control group (PBS), group infected only CA70 (untreated) and groups infected and treated with fluconazole and CAPE (5 mg/Kg). The relative proportion of hemocyte sub-populations in larvae was measured by FACS analysis (*p* is relative to the hemocyte sub-population in the control (PBS) in relation to others groups). ANOVA test (***p < 0.0001).

To explore the immunomodulatory action of CAPE on the cellular immune response of *G. mellonella*, we characterized the hemocyte population by flow cytometry. The hemocyte population was differentiated based on the size and granularity of cells, and at least 5 distinct sub-populations, labeled P1, P2, P3, P5 and P7, were visible (**Figure 6A**). Larvae infected with *C. albicans* showed a significant increase (3.33- fold) in the relative abundance of P1 hemocytes (small and granular cells) compared to control group (PBS +PBS) (*p* < 0.0001). There was a significant increase in hemocyte P5 (very large and nongranular cells) in all groups, except in larvae infected and treated by CAPE. In relation to P7 sub-population, the relative abundance was similar among the groups studied. Interestingly, the sub-populations of P2 and P3 (medium size and granular cells), that are considered important phagocytic cells against pathogens, were significantly increased by the treatment with CAPE in both infected and not infected larvae. When CAPE was administered in *G. mellonella* larvae not infected by *C. albicans*, the P2 and P3 sub-populations increased by 2.2 and 2.47 -fold, respectively, in comparison to control group. In larvae infected by *C. albicans*, the treatment with CAPE reached greater increases in P2 and P3 sub-populations with 2.85 and 17.20-fold, respectively, in relation to infected larvae and untreated (**Figure 6B**). Therefore, CAPE showed a promising action for recruiting phagocytic cells against *C*. *albicans*.

**Figure 7:**
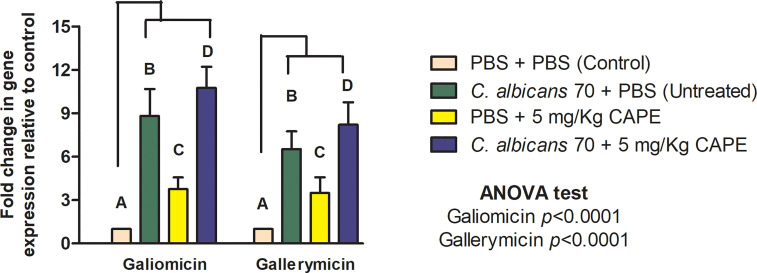
CAPE increased the expression of antimicrobial peptides genes of *G. mellonella* at 24h after treatment of CAPE. Relative quantification (log) of *galiomicin* and *gallerimycin* for the groups inoculated with PBS (Control), infected with CA70 only (untreated), treated with CAPE, and infected and treated with CAPE [CA70 + CAPE (5 mg/Kg)]. Gene expression was represented as mean and SD. Normalization was done using *β-actin* gene. Different letters A, B, C and D represent statistically significant differences among the groups. ANOVA and Tukey Tests (*p* < 0.05).

Subsequently, we investigated the immunomodulatory effects of CAPE on humoral immune response of *G. mellonella* by analyzing the gene expression of antifungal peptides (*galiomycin* and *galeriomycin*). We found that CAPE was able to increase the expression of both *galiomicin* and *galeriomycin* peptides. The group infected with *C. albicans* and treated with CAPE had a statistically significant increase (*p* < 0.0001) in relation to the group infected and untreated (*galiomicin*: 1.93-fold increase; *galeriomycin*: 1.7-fold increase). CAPE induced an increase in the gene expression of *galiomycin* and *galleriomycin* by 3.75 and 3.48-fold, respectively, compared to the control group formed by consecutive PBS injections (**Figure 7**).

**Figure 8:**
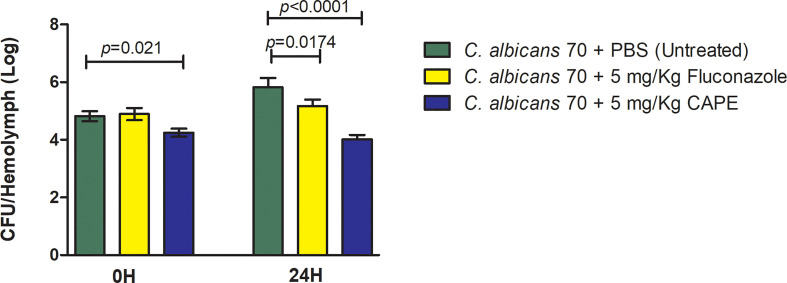
CAPE decreased the number of fungal cells in *G. mellonella* hemolymph. Mean and SD of *C. albicans* counts (CFU/larvae) in the hemolymph of *G. mellonella* at 0 h and 24 h after treatment of CAPE. The following groups were compared at each time of infection: CA70 + PBS (untreated), CA70 + fluconazole and CA70 + CAPE (5 mg/Kg). Treatment with CAPE was able to significantly reduce fungal burden in times evaluated, and fluconazole only after 24 hours. Student’s t-test, *p* < 0.05.

Finally, the antifungal effect of CAPE on experimental candidiasis in *G. mellonella* model was evaluated by the fungal load analysis in the hemolymph. The immediate action of CAPE on infected larvae caused a reduction of 0.58 Log in the CFU/mL in the group infected and untreated (*p* = 0.021). After 24 h, the CAPE treatment was more effective, further reducing the fungal load by 1.80 log compared to the infected and untreated (*p* < 0.0001). In the treatment with fluconazole, only slight reductions in the fungal load were obtained in comparison to the control in 24 h (**Figure 8**). Based on the results obtained in the studies with *G. mellonella* model, CAPE proved to be effective to treat experimental candidiasis by both antifungal activity and immunomodulatory action.

### Evaluation of the Treatment With CAPE on Oral Candidiasis in Mice

**Figure 9:**
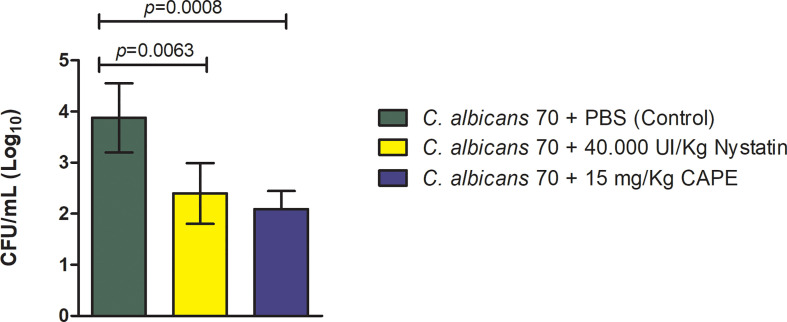
Recovery of *C. albicans* from the oral cavity of mice. *C. albicans* CFU/mL recovered from the oral cavity of mice infected by CA70 and treated with PBS (control group), with nystatin and CAPE (Student’s *t* test, *p* < 0.05).

With antifungal and immunomodulatory CAPE activity demonstrated in the results in *G. mellonella* model, we employed a more refined model of oral candidiasis in mice to continue our investigation of CAPE treatment. *C. albicans* cells were recovered from the oral cavity of all infected mice. The CFU/mL counts were 3.97 ± 0.67 (Log_10_) for the infected and untreated group, 2.39 ± 0.59 (Log_10_) for the group treated with nystatin and 2.0 ± 0.35 (Log_10_) for the group treated with CAPE. Both treatments were capable of reducing *C. albicans* colonization with a statistically significant difference in relation to the untreated group (**Figure 9**).

**Figure 10:**
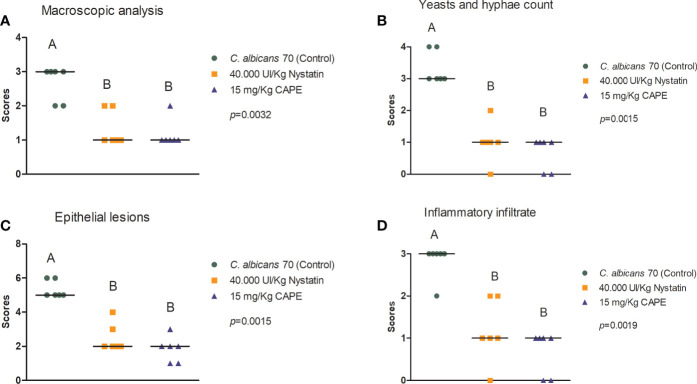
Macroscopic and histological assessment of candidiasis lesions formed on mice’s dorsum tongue. Mice were treated with PBS (control group), nystatin or CAPE **(A–D)**. Scores and median of macroscopic lesions quantification **(A)**, Yeasts count and hyphae in PAS stained sections **(B)**, and epithelial lesions **(C)** and inflammatory infiltrate **(D)** determined in H&E slides (Kruskal-Wallis and Dunn’s posttest; statistically significant differences between groups are represented by different letters).

**Figure 11:**
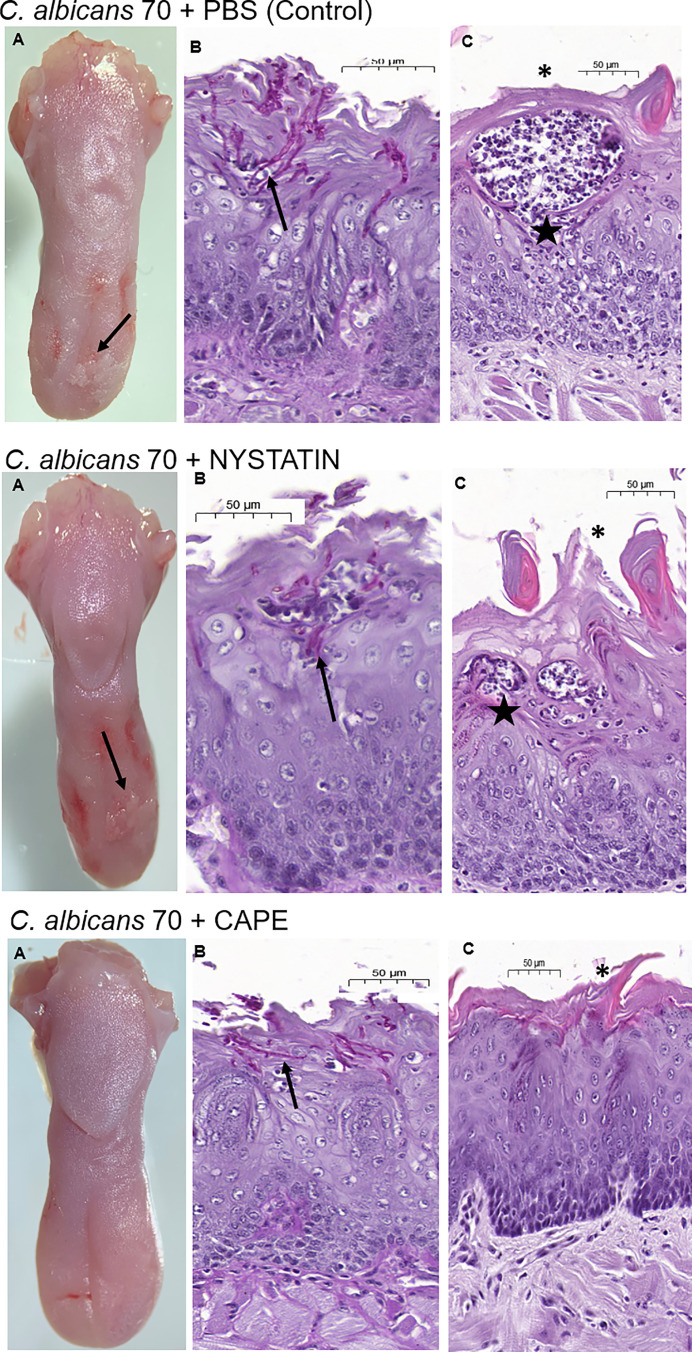
Analysis of the dorsum tongue of mice infected with *C. albicans*. The mice were treated with PBS (control group, top row), nystatin (middle row) and CAPE (bottom row). **(A)** White patches of candidiasis (→) can be observed in the macroscopic analysis of the untreated group and the one treated with nystatin. **(B)** PAS sections, showing hyphae and yeast in the epithelium-keratinized layer (→). **(C)** Images of histological cuts stained by H&E, where intraepithelial microabscesses (☆) and loss of filiform papillae were observed (_*_).

Macroscopic analysis of the dorsum tongue revealed the formation of candidiasis lesions characterized by whitish regions with the presence of pseudomembrane. Promisingly, these lesions were significantly reduced in the groups treated with nystatin or CAPE compared to infected and untreated mice (**Figure 10A**). These findings were confirmed in the microscopic analysis, in which the group treated with CAPE or nystatin presented less quantity of yeasts and hyphae in the keratin layer (**Figure 10B**), of epithelial lesions such as microabscesses, exocytosis, spongiosis and loss of filiform papillae (**Figure 10C**), and of inflammatory infiltrate than infected and untreated group (**Figure 10D**). In general, CAPE was capable of decreasing the macroscopic and microscopic lesions of oral candidiasis (**Figure 11**).

**Figure 12:**
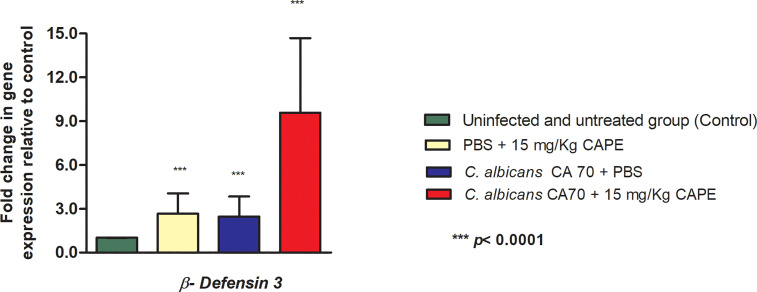
CAPE increased the expression of *β-defensin 3* gene. Relative quantification (log) of *β-defensin 3* gene for the uninfected and untreated group (control), infected with CA70 only, treated with CAPE, and infected with CA70 + CAPE (15 mg/Kg). Gene expression was represented as mean and SD. Normalization was done using *β-actin* gene. ANOVA and Tukey Test (***p < 0.0001).

The immunomodulatory effect of CAPE was also evaluated in the oral cavity of mice by gene expression analysis of *β-defensin 3*. In experimental candidiasis in mice treated with CAPE, this gene showed an increase of 3.91-fold compared to the infected and untreated group. Furthermore, uninfected and CAPE-treated mice, the expression of the *β-defensin 3* gene was increased by 2.66-fold compared to the control group (uninfected and untreated) (**Figure 12**).

Taken together, the study using the oral candidiasis mouse model demonstrated that CAPE was able to reduce the oral colonization by *C. albicans*, to decrease macroscopic and microscopic of candidiasis lesions, and to stimulate the innate defense mechanism associated to β-defensins.

## Discussion

In this study, we investigated the antifungal action of CAPE as a new natural source for the development of therapeutic agents. Mature biofilm was disrupted by the presence of CAPE. Interestingly, the most pronounced effects were observed when CAPE exerted immunomodulatory protection against *C*. *albicans* in two *in vivo* infection models: *G. mellonella* and oropharyngeal candidiasis of immunosuppressed mice. Delivery of the natural compound was found to elevate antifungal peptide expression in the *G. mellonella* model that resulted in prolonged survival. In the murine oral candidiasis model, a significant reduction in fungal cells at the infection site was observed that resulted in reduced lesions and tissue inflammation.

In planktonic assays to determine the antifungal activity of CAPE, we found MIC results ranged from 16 to 64 μg/mL among the 40 clinical *C. albicans* isolates from oral candidiasis. Although inhibition was not as efficacious as standard therapeutic agents, interestingly, CAPE was active against both fluconazole-sensitive and fluconazole-resistant strains, in agreement with some studies that found the same range of MIC (Breger et al., 2007; Coleman et al., 2016; Sun et al., 2018). Sun et al. (2018) found the same MIC values for CAPE (32 to 64 μg/mL) when testing clinical isolates of *C. albicans*, sensitive and resistant to fluconazole, from Shandong Provincial Hospital Qianfoshan. They observed a synergically action of CAPE and fluconazole against resistant clinical isolates of *C. albicans*. Additionally, the CAPE-Fluconazole combination increased the longevity and decreased fungal burden in *C. elegans* when compared with the compounds alone. Some limitations of our study is related to susceptibility of these strains to other antifungals, including echinocandins. In addition, the MIC values for fluconazole, amphotericin B and CAPE that inhibited 50% of growth have not been evaluated.

Since *C. albicans* can be found in both planktonic and biofilm forms during infection, the antifungal effects of CAPE were tested on *C. albicans* biofilms. The elevate resistance to antifungal drugs, the ability to withstand host immune defenses, and the role as a reservoir for continuing infections, makes *Candida* biofilms a crucial factor for the increase morbidity and mortality of patients with *C. albicans* infections (Liu et al., 2017). In our study, biofilms treated with CAPE at a concentration of 5 x MIC showed significant reductions in the viable cell count (CFU/mL) and in the total biomass. Biofilms treated with CAPE reached reductions of up to 68.5% in the amount of total biomass in relation to the untreated control group. Likewise, some studies have shown the antifungal effect of CAPE on *C. albicans* biofilms as an alternative in the treatment or prevention of this infection (De Vita et al., 2014; Freires et al., 2016). The reduction in the biomass of the biofilm obtained in this study were superior to those found by (Breger et al., 2007), who tested the effects of CAPE on mature biofilms formed by the *C. albicans* DAY185 in silicone squares. They found a 50% reduction in the dry weight of CAPE-treated biofilms compared to untreated control.

Although fluconazole has few side effects, its extensive application has led to the frequent emergence of resistance compromised the successful treatment of oral candidiasis. Treatment with fluconazole at concentration of 5x MIC (320 μg/mL) was not so effective as CAPE in reducing the total biomass of *C. albicans* biofilms (only 32% of reduction). This result was expected since the *C. albicans* strain employed in our study showed resistance to fluconazole in MIC assays. Several studies have reported low susceptibility of biofilm-producing *C. albicans* strains to fluconazole, a front-line antifungal drug for the treatment of oral candidiasis (Mukherjee et al., 2003; Uppuluri et al., 2008; Jia et al., 2016; Lu et al., 2019). Fluconazole showed an insignificant inhibition (< 20%) of *C. albicans* biofilms at concentration of 1024 μg/mL (Jia et al., 2016).

Microscopic images confirmed CAPE impacted *C*. *albicans* biofilm, reducing the number of recovered fungal cells and overall biomass. Importantly, this analysis showed that treatment with CAPE inhibited the hyphae formation that is another crucial virulence factor of *C. albicans* species. Here, we also analyzed the gene expressions of *C. albicans* in biofilms employing several genes associated with different virulence mechanisms. Genes related to surface adhesins, such as *ALS1* (agglutinin-like sequence) and *YWP1* (yeast cell wall protein) were suppressed by CAPE treatment in biofilms. It is known that the agglutinin-like sequence (*ALS*) genes play an important role in *C. albicans* adhesion and biofilm formation on biotic and abiotic surfaces (Hosseini et al., 2019; Jordão et al., 2021). *YWP1* might be involved in *C. albicans* yeast dispersion, once it was found that *YWP1*-deficient blastoconidia exhibited increase adhesiveness and biofilm formation (Granger et al., 2005). Therefore, the decrease in the expression of *ALS1* and *YWP1* represent a promising action of CAPE in control to prevent *C. albicans* biofilm formations.

Gene expression analysis revealed a decrease in the expression of an integrated network of transcription factors *(BCR1, BGR1, CPH1, EFG1, NDT80, ROB1, TEC1 and UME6*) that play a role in regulating biofilm-related processes. Nobile et al. (2012) have provided a portrait of a six-transcription factors network (*BRG1, NDT80, ROB1, TEC1, BCR1 and EFG1*) involved in a variety of phenotypes with relevance to *C. albicans* biology and pathobiology. Their data indicated that the transcriptional circuitry for biofilm formation is highly integrated and regulates the expression of key biofilm effector genes. Apart from biofilm formation, the secreted virulence factors of *C. albicans*, such as proteases and phospholipases, contribute to elevate its pathogenicity. These hydrolases degrade surface membrane proteins, thereby facilitating the invasion of *C. albicans* into host tissue (Mayer et al., 2013). Overall, our results indicate that CAPE treatment may affect biofilm formation by reducing the levels of adhesins and by restricting the long-term maintenance of biofilm through transcriptional regulator factors. In addition, CAPE can impair *C. albicans* invasion into host tissues by inhibiting the expression of proteases and phospholipases. Thereby CAPE exerts its anti-biofilm activity towards *C. albicans* biofilm through a multi-target mode of action, which differs from commonly used antifungals.

Assessment of the impact of CAPE during an active infection using *in vivo* studies immunomodulatory effects. *G. mellonella* is a widely studied infection model, which provides the possibility of evaluating the efficacy of new antimicrobial compounds and immune host responses. *G. mellonella* larvae are easy to use, inexpensive to purchase, and free from the legal/ethical restriction that are applied to the use of mammals (Kavanagh and Sheehan, 2018; Pereira et al., 2018). The use of *G. mellonella* larvae in this capacity will not completely replace the need to use of mammalian models in this role, but judicious use of larvae can accelerate the identification of potential therapeutic doses for use in mammals and allow the rapid initial evaluation of antifungal agents (Kavanagh and Sheehan, 2018). In addition, the larvae immune system presents structural and functional similarities to the innate immune response of mammals, which are composed of cellular (hemocytes) and humoral defenses (antimicrobial peptides) (Pereira et al., 2018; de Barros et al., 2019). The cellular branch of *G. mellonella* immunity is mediated by hemocytes. *G. mellonella* possesses five types of hemocytes: prohemocytes, granulocytes, plasmatocytes, oenocytoids and spherulocytes (Browne et al., 2015; Wojda et al., 2020). To the best of our knowledge, this is the first article in the literature that investigated the anti-*C. albicans* properties of CAPE using *G. mellonella*. We found that the treatment with CAPE in *G. mellonella* larvae infected by *C. albicans* increased the survival rate by killing *C. albicans* cells in hemolymph and also by stimulating the immune system.

The number of hemocytes was increased after treatment with CAPE, mainly the P2 and P3 subpopulations that are granular cells with function in phagocyting pathogens (Araújo et al., 2008; Browne et al., 2015). The increase relative proportion of P2 and P3 hemocytes with the CAPE treatment corresponded to a decrease in the proportion of P5 hemocytes. P5 hemocytes demonstrate globular inclusions and resembled adipohemocytes, which stores energy in the form of lipids and glycogen (Araújo et al., 2008; Browne et al., 2015). The reduction in P5 cells in larval hemocyte population treated with CAPE may be an indication that energy reserves in the form of lipids were used to maintain their survival against infection by *C. albicans* (Browne et al., 2015).

In addition to modulating hemocyte responses, antifungal peptide expression was also altered. The immune response of *G. mellonella* against pathogens is comprised of a range of antimicrobial peptides (AMPs), which are produced according to the aggressor agent type. Thus, we further explored alterations in the immune response examining the expression of antifungal peptides such as *galiomicin*, a defensin, and *gallerymicin*, a cysteine-rich peptide. We observed an increased expression of both AMPs, indicating that CAPE also modulated the humoral immune response of *G*. *mellonella* larvae, which may have contributed to protect larvae from *C. albicans* infection. Taken together, these findings indicate that CAPE is capable of stimulating the cellular and humoral immune responses of the larvae and consequently prevent *C. albicans* infection.

Finally, the effects of CAPE were evaluated in an oral candidiasis murine model which mimics the ones observed in the human oral cavity, such as salivary flow, pH variations, the presence of teeth, mucous membranes characteristics and immune response (Sparber and LeibundGut-Landmann, 2015; Borman, 2018). We verified that the treatment with CAPE in mice infected by *C. albicans* was able to reduce the fungal burden, hyphae invasion and inflammation in the oral tissues compared to untreated animals. A single treatment with CAPE was able to reduce the fungal load in the mouth of mice with oral candidiasis by 1.97 log (CFU/mL).

To investigate the possible mechanisms related to the preventive effects of CAPE on oral candidiasis in mice, we analyzed the gene expression of antimicrobial peptides with local action in the immune defense of oral cavity. Epithelial cells of mice release small anti-microbial peptides (AMPs), especially in saliva, which are capable of exerting direct effects against pathogens, including the family of defensins (Tomalka et al., 2015). Certain murine defensins have shown antifungal properties, such as β-defensin 3 (mBD3), which induces perforations in the fungal wall, and consequently, cell lysis (Jiang et al., 2012; Tomalka et al., 2015). The mBD3 is essential to prevent oropharyngeal candidiasis in mice (Conti et al., 2016), although it has no direct ortholog in humans (Ganz, 2003; Zhou et al., 2020). In this context, CAPE treatment exhibited potent anti-*C. albicans* activity in oral cavity of mice, as it was able to stimulate the expression of the *β-defensin 3* gene, favoring the reduction of candidiasis lesions.

In summary, CAPE exhibited strong antifungal activity against a large number of *C. albicans* oral cavity isolates, including drug resistant strains. CAPE also showed activity against *C. albicans* biofilms, leading to inhibition of viable cells, decrease of the total biomass, reduction of filamentation and downregulation of genes associated with adherence, transcriptional factors and enzymes production. In *in vivo* study, CAPE protected *G. mellonella* larvae from candidiasis due to both antifungal and immunomodulatory properties. The treatment with CAPE increased the larvae survival by killing the fungal cells and by stimulating the humoral and cellular immune responses. In addition, CAPE was able to decrease oral candidiasis in mice with significant reductions of the macroscopic lesions, penetration of hyphae into oral tissues, epithelial damages and inflammatory infiltrate, as well as with an increase in the expression of *β-defensin 3* gene. The treatment with CAPE in *G. mellonella* and mouse models was not harmful to the hosts. Thus, CAPE is a promising natural compound that should be explored further for its potential in prevention and treatment of oral candidiasis.

## Data Availability Statement

The raw data supporting the conclusions of this article will be made available by the authors, without undue reservation.

## Ethics Statement

The animal study was reviewed and approved by Ethics Committee on the Use of Animals of the ICT/UNESP under protocol 019/2019-CEUA-ICT UNESP.

## Author Contributions

Conceived and designed the experiments: JJ and EM. Performed the experiments: PB. Contributed to FACS analysis: VK and FL. Contributed to the experiments in oral candidiasis model: RR and MG. Contributed with reagents/materials/analysis tools in RIH/Brown University: BF and EM. Analyzed the data: PB and JJ. Wrote and revised the paper: PB, BF, EM, and JJ. All authors contributed to the article and approved the submitted version.

## Conflict of Interest

The authors declare that the research was conducted in the absence of any commercial or financial relationships that could be construed as a potential conflict of interest.

## Publisher’s Note

All claims expressed in this article are solely those of the authors and do not necessarily represent those of their affiliated organizations, or those of the publisher, the editors and the reviewers. Any product that may be evaluated in this article, or claim that may be made by its manufacturer, is not guaranteed or endorsed by the publisher.
